# Physical and Genetic Associations of the Irc20 Ubiquitin Ligase with Cdc48 and SUMO

**DOI:** 10.1371/journal.pone.0076424

**Published:** 2013-10-14

**Authors:** Aaron Richardson, Richard G. Gardner, Gregory Prelich

**Affiliations:** 1 Department of Genetics, Albert Einstein College of Medicine, Bronx, New York, United States of America; 2 Department of Pharmacology, University of Washington, Seattle, Washington, United States of America; Texas A&M University, United States of America

## Abstract

A considerable percentage of the genome is dedicated to the ubiquitin-proteasome system, with the yeast genome predicted to encode approximately 100 ubiquitin ligases (or E3s), and the human genome predicted to encode more than 600 E3s. The most abundant class of E3s consists of RING finger-containing proteins. Although many insights have been obtained regarding the structure and catalytic mechanism of the E3s, much remains to be learned about the function of the individual E3s. Here we characterize *IRC20*, which encodes a dual RING- and Snf/Swi family ATPase domain-containing protein in yeast that has been implicated in DNA repair. We found that overexpression of *IRC20* causes two transcription-associated phenotypes and demonstrate that the Irc20 RING domain possesses ubiquitin E3 activity *in vitro*. Two mass spectrometry approaches were undertaken to identify Irc20-associated proteins. Wild-type Irc20 associated with Cdc48, a AAA-ATPase that serves as an intermediary in the ubiquitin-proteasome system. A second approach using a RING mutant derivative of Irc20 detected increased association of the Irc20 mutant with SUMO. These findings provide a foundation for understanding the roles of Irc20 in transcription and DNA repair.

## Introduction

Proteins are often subject to post-translational modification as part of signal transduction pathways to rapidly regulate their activity or abundance in response to changes in the environment. In addition to post-translational modification by small chemical groups such as phosphate, acetyl, or methyl groups, proteins can also be modified by the ubiquitin family of proteins. Ubiquitin is a highly conserved 76-amino acid protein that is covalently attached to substrates either as single ubiquitin moieties or as ubiquitin chains, with a poly-ubiquitin chain typically targeting its substrate for degradation via the 26S proteasome [1]. Conjugation requires C-terminal processing of the ubiquitin precursor, followed by a cascade of E1, E2, and E3 enzymes that successively activate ubiquitin and direct its attachment to the substrate [1]. Approximately ten other ubiquitin-like proteins, including Small Ubiquitin-like MOdifier (SUMO) [2], are conjugated to substrates via similar mechanisms, although each pathway has its own dedicated set of enzymes. The functions of SUMO and the other ubiquitin-like proteins are distinct from that of ubiquitin, with frequent cross-pathway regulation or interactions being uncovered [3], [4].

As the final enzyme in the conjugation pathway, the identification of ubiquitin ligase (or E3s) and understanding their substrate specificity, regulation, and functions remains a major goal of ubiquitin research. Three groups of E3s exist, each distinguishable by their sequence motifs and conjugation mechanism. RING and U-box E3s facilitate the transfer of ubiquitin from the E2 to the target by functioning as a scaffold or adaptor that recruits the E2 and substrate together [5], [6], whereas HECT E3s form a thioester bond with activated ubiquitin, which is subsequently transferred from the HECT active cysteine residue directly onto the target substrate [7]. Most E3s can polymerize chains onto substrates by ligating additional ubiquitin monomers onto any one of seven lysine residues of a previously conjugated ubiquitin [8], [9]. A final class of ubiquitin ligases, E4s, can only ligate ubiquitin onto other ubiquitin residues and therefore requires a pre-conjugated residue to generate a ubiquitin chain on a substrate protein [10].

Ubiquitylation has multiple functions, with its most common role being the targeting of the modified substrate to the proteasome for degradation. For some proteins, however, conjugation to ubiquitin is not sufficient to target them to the proteasome. One of the key intermediaries in the pathway is Cdc48/p97, a highly conserved essential AAA-ATPase that bridges ubiquitylation and proteasomal degradation [11] by using its “segregase” activity to extract a ubiquitylated substrate from membranes or from protein complexes [12], [13]. Cdc48/p97 is required for diverse biological processes including endoplasmic reticulum-associated degradation (ERAD) [14], [15], processing of the transcription factor Spt23 [13], [16], and the proteolytic ubiquitin fusion degradation (Ufd) [11] and N-end rule [17] pathways. Cdc48 participates in these diverse pathways through its intrinsic ubiquitin-binding activity [18] and by associating with an array of co-factors [19], including ubiquitin-binding proteins that recruit conjugated proteins to Cdc48 [19], multiple RING finger E3s such as Ufd2 [10], Hrd1, and Doa10 [20], and a de-ubiquitinating enzyme (Ufd3) that regulate chain length [21]. Through this association with a wide variety of co-factors, Cdc48 is involved in a diverse array of cellular processes. The substrate recruitment factors typically bind to the Cdc48 N-terminal domain [22] while the Ufd2 and Ufd3 substrate processing factors bind at the Cdc48 C-terminus [21]. In this way, Cdc48 acts as a scaffold that brings a target protein together with ubiquitin pathway enzymes that direct the degree of ubiquitylation and ultimately the fate of the substrate.

One of the prominent roles of the ubiquitin pathway is the regulation of transcription factors. The effects on transcription occur at many levels, ranging from affecting chromatin structure via mono-ubiquitylation of histone H2B [23]–[25], causing degradation of RNA polymerase II in response to UV damage [26], processing of transcription factors such as Spt23 [13] and SREBP [27], recycling or licensing of site-specific DNA-binding proteins such as Gal4, [28] and dissociation of the α2 transcriptional repressor from its target promoters [29]. Our lab has been using a genetic approach to identify transcriptional regulators in *Saccharomyces cerevisiae*, screening for mutations that increase transcription from the UAS-less *suc2Δuas(−1900/−390)* reporter [30], [31]. This Bur- (Bypass UAS Requirement) selection has been very successful, revealing mutations in genes that regulate TATA-Binding Protein, RNA polymerase II, and histones [30], [32], [33]. A yeast strain containing the *suc2Δuas(−1900/−390)* reporter was subsequently screened for genes whose overexpression causes in the Bur- phenotype, resulting in the isolation of a single gene, *IRC20* [34].

*IRC20* (Increased Recombination Centers 20) was identified originally in a screen for gene deletions that increase the spontaneous formation of nuclear Rad52 foci as a marker for non-homologous DNA repair [35]. A connection to DNA repair is further supported by the observation that *irc20Δ* decreases the frequency of synthesis-dependent strand-annealing (SDSA) events and homologous chromosome crossovers and suppresses the activity of the DNA repair genes, *SRS2* and *MRE11* [36]. In humans, the genes most similar to *IRC20* are SHPRH and HLTF, two RING finger-containing ATPases that maintain genomic stability via the polyubiquitination of proliferating cell nuclear antigen (PCNA) at stalled replication forks [37].

Irc20 contains two predicted domains: a Snf2/Swi2 family ATPase/helicase domain that is a common characteristic of proteins involved in chromatin remodeling [38] and a C3HC4 domain that characterizes the RING subset of E3s [39]. Here we demonstrate that Irc20 has E3 activity *in vitro*, identify domains needed for its function *in vivo*, and find that Irc20 physically associates with Cdc48 and is modified by SUMO. Our results constitute a step towards the goal of understanding the *in vivo* function of this gene.

## Materials and Methods

### Strains, plasmids, and media

*S. cerevisiae* strains used in this study are listed in Table 1 and plasmids constructed for this study are listed in Table 2. All media used, including rich media (YPD) and synthetic complete drop-out media (for example, SC–Ura) were made as described [40]. YPsuc plates contained 2% sucrose and 1 μg/ml antimycin A. SC+Gal plates were synthetic complete (SC) media containing 2% galactose. 5-Fluoroorotic acid (5-FOA) sensitivity was assayed on plates containing 1 g/L 5-FOA. Activation of the *suc2Δuas(−1900/−390)-HIS3* reporter was assayed on SC-His plates containing 0.1 mM 3-Amino-1,2,4-triazole (3-AT) and 0.2 mM 3-AT. G418 resistance was assayed on YPD containing 200 μg/ml gentimicin (Gibco 15750–060). Standard genetic methods for mating, sporulation, transformation, and tetrad analysis were used throughout this study [40]. pGP764 (2 µ *TRP1 GAL4(DBD)-6xSMT3*) was generated by PCR amplification of an *SMT3* cassette lacking the C-terminal di-glycine motif. The *SMT3* cassette was subcloned into pGBKT7 and the insert was elongated to a 6×SUMO chain through consecutive rounds of subcloning.

**Table 1:** *S. cerevisiae* strains used in this study.

Strain	Genotype
GY460	*MATa his4–912δ lys2–128δ suc2Δuas(−1900/−390) ura3–52 leu2Δ1*
GY480	*MATa his4–912δ lys2–128δ suc2Δuas(−1900/−390) ura3–52 leu2Δ1 trp1Δ63*
GY482	*MATa his4–912δ lys2–128δ ura3–52 leu2Δ1 trp1Δ63*
GY2171	*MATa/α his3Δ200?his3Δ200 lys2–128δ/lys2–128δ suc2Δuas(−1900/390)-HIS3/suc2Δuas(−1900/−390)-HIS3 ura3–52/ura3–52 trp1Δ63/trp1Δ633 leu2Δ1/leu2Δ1*
GY2203	*MATa his4–912δ lys2–128δ ura3–52 leu2Δ1 trp1Δ63 GAL1pr-TAP-IRC20::TRP1*
GY2205	*MATa his4–912δ lys2–128δ suc2Δuas(−1900/−390) ura3–52 leu2Δ1 trp1Δ63 TAP-IRC20*
GY2333	*MATα his4–912δ lys2–128δ suc2Δuas(−1900/−390) leu2Δ1 trp1Δ63 CDC48–3xMYC::k.i.TRP1*
GY2334	*MAT*a *his4–912δ lys2–128δ suc2Δuas(−1900/−390) ura3–52 leu2Δ1 trp1Δ63 CDC48–3xMYC::k.i.TRP1*
GY2385	*MATα his3Δ200 lys2–128δ suc2Δuas(−1900/390)-HIS3 ura3–52 trp1Δ63 leu2Δ1cdc48Δ::TRP1 <pAR54>*
GY2387	*MATα his3Δ200 lys2–128δ suc2Δuas(−1900/390)-HIS3 ura3–52 trp1Δ63 leu2Δ1cdc48Δ::TRP1 <pAR56>*
GY2406	*MATα his3Δ200 lys2–128δ suc2Δuas(−1900/390)-HIS3 ura3–52 trp1Δ63 leu2Δ1cdc48Δ::TRP1 <pAR64>*
GY2433	*MATα his3Δ200 lys2–128δ suc2Δuas(−1900/390)-HIS3 ura3–52 trp1Δ63 leu2Δ1cdc48Δ::TRP1 <pAR73>*
GY2441	*MATa his3Δ200 lys2–128δ suc2Δuas(−1900/390)-HIS3 ura3–52 trp1Δ63 leu2Δ1cdc48Δ::TRP1 irc20Δ::KANMX <pAR65>*
GY2443	*MATa his3Δ200 lys2–128δ suc2Δuas(−1900/390)-HIS3 ura3–52 trp1Δ63 leu2Δ1cdc48Δ::TRP1 irc20Δ::KANMX <pAR75>*
GY2476	*MATa/α his4–912δhis4–912δ lys2–128δ/lys2–128δ suc2Δuas(−1900/390)/suc2Δuas(−1900/−390) ura3–52/URA3 trp1Δ63/trp1Δ63 leu2Δ1/lLEU2 CDC48/CDC48-9xMYC::KANMX*
OY382	*MATa his4–912δ lys2–128δ suc2Δuas(−1900/−390) leu2Δ1 ubc4Δ::KANMX*
OY405	*MATa his4–912δ lys2–128δ suc2Δuas(−1900/−390) leu2Δ1 ura3–52 ubc8Δ::KANMX*
OY463	*MATa his3Δ1 lys2–128δ suc2Δuas(−1900/−390) leu2Δ1 met15Δ0 ubc2Δ::KANMX*
OY464	*MATα his3Δ1 lys2–128δ suc2Δuas(−1900/−390) leu2Δ1 met15Δ0 ubc5Δ::KANMX*
OY466	*MATa his3Δ1 lys2–128δ suc2Δuas(−1900/−390) leu2Δ1 met15Δ0 ura3Δ0 ubc7Δ::KANMX*
OY467	*MATα his3Δ1 lys2–128δ suc2Δuas(−1900/−390) leu2Δ1 met15Δ0 ubc10Δ::KANMX*
OY468	*MATa his3Δ1 lys2–128δ suc2Δuas(−1900/−390) leu2Δ1 met15Δ0 ura3Δ0 ubc11Δ::KANMX*
OY470	*MATa his3Δ1 lys2–128δ suc2Δuas(−1900/−390) leu2Δ1 met15Δ0 ura3Δ0 ubc13Δ::KANMX*
OY471	*MATa his3Δ1 lys2–128δ suc2Δuas(−1900/−390) leu2Δ1 met15Δ0 ura3Δ0 mms2Δ::KANMX*

**Table 2:** Plasmids used in this study.

pAR6	KAN *GST-IRC20-RING*
pAR7	AMP 2μ *LEU2 IRC20*
pAR9	AMP 2μ *LEU2 IRC20–6xHA*
pAR11	AMP 2μ *LEU2 TEFpr-3xHA-IRC20*
pAR14	AMP 2μ *LEU2 TEFpr-3xHA-irc20-C1239A*
pAR16	AMP 2μ *URA3 TEFpr-3xHA-IRC20*
pAR21	KAN *GST-irc20-RING-C1239A*
pAR30	AMP 2μ *LEU2 TEFpr-3xHA-irc20-D534A E535A*
pAR35	AMP 2μ *LEU2 GAL1pr-TAP-IRC20*
pAR41	AMP 2μ *LEU2 TAP-IRC20*
pAR52	AMP 2μ *LEU2 CDC48*
pAR54	AMP CEN *URA3 CDC48*
pAR55	AMP 2μ *URA3 CDC48*
pAR56	AMP CEN *LEU2 CDC48*
pAR58	AMP 2μ *URA3 IRC20 (a.a. 1–1556)*
pAR59	AMP 2μ *URA3 IRC20 (a.a. 1–1238)*
pAR60	AMP 2μ *URA3 IRC20 (a.a. 1–386)*
pAR61	AMP 2μ *URA3 IRC20 (a.a. 584–1238)*
pAR62	AMP 2μ *URA3 IRC20 (a.a. 584–1556)*
pAR64	AMP CEN *LEU2 cdc48-R369K*
pAR65	AMP CEN *URA3 CDC48–3xMYC*
pAR67	AMP 2μ *URA3 IRC20 (a.a. 584–882)*
pAR68	AMP 2μ *URA3 IRC20 (a.a. 883–1033)*
pAR69	AMP 2μ *URA3 IRC20 (a.a. 1034–1238)*
pAR70	AMP 2μ *URA3 IRC20 (a.a. 584–1033)*
pAR71	AMP 2μ *URA3 IRC20 (a.a. 904–1238)*
pAR73	AMP CEN *URA3 cdc48-R369K*
pAR75	AMP CEN *URA3 cdc48-R369K-3xMYC*
pAR84	AMP 2μ *URA3 irc20 (Δ684–882)*
pAR85	AMP 2μ *URA3 irc20 (Δ883–1033)*
pAR86	AMP 2μ *URA3 irc20 (Δ1034–1238)*
pAR87	AMP 2μ *URA3 irc20 (Δ883–904)*
pGBKT7	KAN 2μ *TRP1 GAL4(DBD)*
pGM7	AMP 2μ *LEU2 IRC20*
pGP740	KAN 2μ *TRP1 GAL4(DBD)-SMT3*
pGP764	KAN 2μ *TRP1 GAL4(DBD)-6xSMT3*
pRS416	AMP CEN *URA3*
pRS425	AMP 2μ *LEU2*
pRS426	AMP 2μ *URA3*
YGPM-29e18	KAN 2μ *LEU2 Chr.IV 235592–245652*
YGPM-4d03	KAN 2μ *LEU2 Chr.IV 237368–247567*
pZW61	AMP 2μ *URA3 SMT3*

### Generation of RING finger and ATPase mutants

Agilent's Quikchange XL Site-Directed Mutagenesis Kit (#200517) was used with primers GO1653 (5′ ATTAAACGATAATCAAATATTGAGCGCCTCTATCTGTTTGGGAGAAGTTGAA 3′) and GO1654 (5′ TAATTTGCTATTAGTTTATAACTCGCGGAGATAGACAAACCCTCTTCAACTT 3′) to introduce a TGC to GCC change on plasmid pAR7, which replaces the cysteine with an alanine at amino acid position 1239 in the RING finger domain of Irc20 and generating plasmid pAR8. Primers GO1870 (5′ TGCAGTTTTATAGAATCATTCTGGCTGCAGTTCAAATGCTACGTAGTTCATC 3′) and GO1871 (5′ GATGAACTACGTAGCATTTGAACTGCAGCCAGAATGATTCTATAAAACTGCA 3′) were used to introduce a GATGAA to GCTGCA change in plasmid pAR16, which replaces adjacent aspartic acid and glutamic acid residues each with alanine residues.

### *In vitro* ubiquitylation assay

Lysates were prepared from BL21(DE3)-RIPL bacteria transformed with plasmids encoding either GST-Irc20-RING (pAR6), GST-Irc20-C1239A RING (pAR21), or GST only (pGEX-KG), grown to saturation overnight at 37°C in 5 ml LB media treated with 100 μg/ml ampicillin and 50 μg/ml chloramphenicol. 150 μl of the overnight culture was used to inoculate each of two tubes (“induced” and “uninduced”) containing 5 ml of fresh LB media without antibiotics and shaken at 37°C for 3 hours. Expression for one of the two cultures from each strain was induced by adding 50 μl of 0.1 M IPTG and shaken at 37°C for one hour. Cultures were pelleted, transferred to a 1.5 ml microfuge tube, resuspended in 300 μl cold PBS + protease inhibitors, and sonicated on ice for 10 seconds at sonication level #5. Cell debris was pelleted by centrifuge at maximum speed for 5 minutes (4°C) and the supernatant was transferred to a fresh 1.5 ml microfuge tube. Total protein concentration was measured by Bradford dye assay. *In vitro* ubiquitylation assay reactions were assembled on ice containing 100 ng of purified Uba1, 200 ng of purified human UbcH5a, 50 mM Tris-Cl (pH 7.3), 2.5 mM MgCl_2_, 500 μM DTT, 2 mM ATP, and 10 μg of total protein from lysates prepared from bacteria expressing either GST-RING (pAR6), GST-ringC1239A (pAR21), or GST only (pGEX-KG). Reactions were incubated at 30°C for 90 minutes and then subject to electrophoresis on a 10% SDS-PAGE gel for western blot analysis. Westerns were probed with either anti-Ub (Enzo Life Sciences UG 9510) or anti-GST (from Dr. Peter Davies) antibodies.

### Preparation of yeast protein extracts

For immunoprecipitation, yeast cultures were grown to an A_600_ of 1.0 and harvested by centrifugation. Pellets were frozen, resuspended in RNP lysis buffer (0.1 M HEPES pH 7.4, 100 mM NaCl, 0.1% NP-40, 0.1 mM PMSF, 1 mg/ml each of leupeptin, pepstatin, and aproptinin) and lysed by vortexing in the presence of glass beads. Cell debris was pelleted by centrifugation and cleared lysates collected into a fresh tube. For cell lysis in SUMO co-IPs, 10 mM N-Ethylmaleimide (NEM) (Sigma E3876) was added to RNP buffer.

### Immunoprecipitations and western blot analysis

Immunoprecipitations were conducted by adding 1 μg crude cell extract to 40 μl anti-HA conjugated agarose beads (Sigma E6779-1ML) in a volume of 0.6 ml and rotated at 4°C for 90 minutes. Beads were washed 3 times with 500 μl RNP buffer and the protein eluted by boiling for 2 minutes. N-terminal HA-tagged wild-type Irc20, RING and ATPase point mutants, and Irc20 deletion derivatives were detected with anti-HA antibody (Covance #MMS-101R-500). TAP-Irc20 was detected with Peroxidase Anti-Peroxidase (PAP) Soluble Complex antibody (Sigma P1291). Cdc48-3xMYC fusion protein was detected with anti-MYC antibody (Invitrogen 46–0603). Untagged SUMO was detected with anti-SUMO antibody (from Dr. Steve Brill and Dr. Pamela Meluh). Gal4(BDB)-SUMO fusion proteins were detected with anti-GAL4(DBD) antibody (Santa Cruz Biotechnology sc-577. An anti-G6PDH antibody (Sigma #A9521) was used as a loading control for co-IPs.

### Mass spectrometry

For mass spectrometry performed under native lysis conditions, three 1L SC-Leu cultures of GY460 transformants carrying either pGM7 (2 μ *LEU2 IRC20*) or pAR11 (2 μ *LEU2 TEF2pr-3xHA-IRC20*) were grown and lysed as described above. For each biological replicate, immunoprecipitations were performed by adding 15 mg of total protein lysates to 50 μl anti-HA agarose beads (Santa Cruz Biotechnology #sc-7392 AC) at 4°C for 2 hours. Protein was eluted from the beads by incubating at 4°C for 2 hours with 500 μg HA peptide (Roche #11666975001) and eluates were concentrated by TCA precipitation.

For the *in vivo* crosslinking experiments, GY460 transformants carrying either pGM7 (2 μ *LEU2 IRC20)*, pAR11 (2 μ *LEU2 TEF2pr-3xHA-IRC20*), or pAR14 (2 μ *LEU2 TEF2pr-3xHA-irc20-C1239A*), were each grown in three 100 ml SC-Leu cultures to an A_600_ of 1.0, crosslinked with 1% formaldehyde for 9 minutes, quenched with 125 mM glycine, and frozen for 5 minutes in liquid nitrogen. Cell lysis and immunoprecipitations were performed as previously described [41].

### Creation of chemically mutagenized plasmid library and selection for *cdc48* Bur- mutants

10 μg of a *LEU2*-marked CEN plasmid carrying *CDC48* (pAR56) was treated for 90 minutes with 500 μl of hydroxylamine solution pH 7.0 (1M hydroxylamine, 450 mM NaOH). The reaction was stopped by adding 500 μl of mutagenesis stop solution (20 mM NaCl, 50 mg BSA in 95% ethanol) and incubated on ice for 30 minutes. The DNA was recovered by ethanol precipitation and resuspended in TE to a concentration of ∼100 ng/μl.

*CDC48* Bur- mutants were isolated by a standard plasmid shuffle strategy by transforming a *cdc48Δ::TRP1* <*CEN URA3 CDC48*> strain (GY2385) with the library of mutagenized plasmids and plating onto SC-L plates. Transformants were replica plated onto SC-LH + 5FOA to select for those colonies that lost the *URA3*-marked *CDC48* plasmid yet carried a mutagenized *LEU2*-marked plasmid that was capable of activating transcription from the *suc2Δuas(−1900/−390)-HIS3* reporter.

## Results

### Overexpression of *IRC20* causes transcription phenotypes

**Figure 1:**
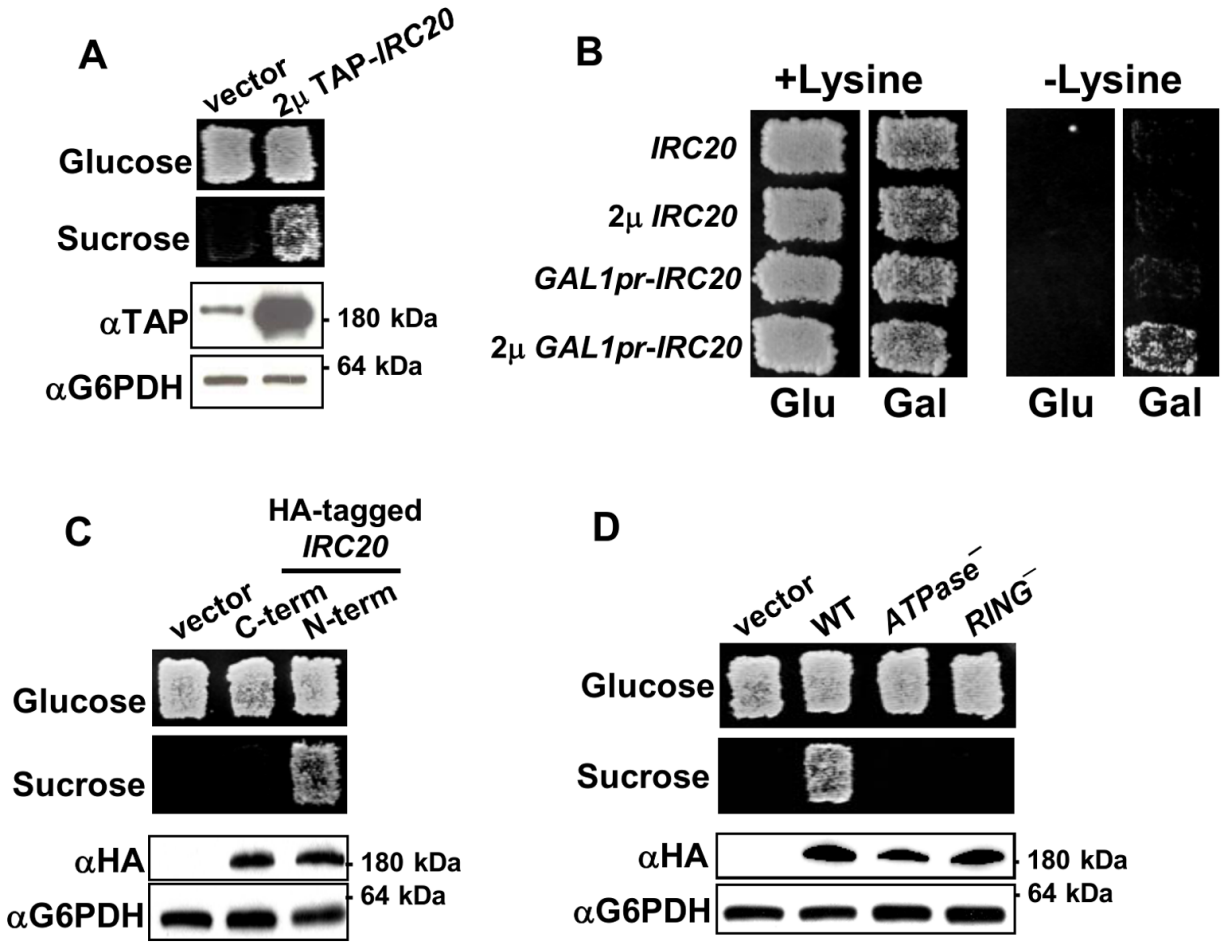
Overexpression of *IRC20* causes transcription phenotypes. A) Yeast strain GY2205, which contains the *suc2Δuas(−1900/−390)* reporter and endogenous *TAP-IRC20*, was transformed with either empty vector (pRS425) or a 2 μ *TAP-IRC20* plasmid (pAR41) and assayed for the Bur phenotype on a YPsucrose plate (top). A western blot to detect expression of TAP-Irc20 is shown below, along with G6PDH loading control. B) 2 μ *GAL1pr-IRC20* exhibits an Spt- phenotype. Yeast strains GY482 (*his4–912δ lys2–128δ*) and GY2203 (*his4–912δ lys2–128δ GAL1pr-TAP-IRC20*) were transformed with the indicated plasmids and transformants were assessed for their Spt phenotype on SC-His and SC-Lys plates. C) Overexpression of epitope-tagged Irc20. Irc20 was HA-tagged at its N- or C-terminus and expressed from a 2 μ plasmid. The 2 μ Bur- phenotype was disrupted by the C-terminal 6HA tag (pAR9), while N-terminal 3xHA-Irc20 fusion (pAR11) remained functional. D) N-terminally HA-tagged D534A E535A *irc20* ATPase (pAR30) and C1239A RING finger (pAR14) mutants were created and expressed from a 2 μ plasmid in yeast strain GY460. Both mutants are expressed at similar levels as wild-type Irc20, but are incapable of producing the 2 μ Bur- phenotype.

The Bur selection was designed to identify general transcriptional regulators by selecting for mutations that increase transcription from the UAS-less *suc2Δuas(−1900/−390)* reporter [30]. As predicted, all of the loss-of-function mutations identified in this selection were in genes that have relatively broad roles as transcriptional regulators [30], [32], [33]. To identify genes whose gain of function causes a Bur- phenotype, a yeast strain was transformed with a systematic library of yeast 2 μ plasmids and screened for genes whose overexpression increases transcription of *suc2Δuas(−1900/−390)* [34]. A single plasmid was obtained, and subcloning revealed that overexpression of *IRC20* was responsible for the Bur- phenotype. *IRC20* encodes a 1,556 amino acid protein that contains a RING domain and a Snf/Swi family ATPase/helicase domain. Irc20 was TAP-tagged at its N-terminus and shown to be functional based on its ability to activate the *suc2Δuas* reporter, resulting in growth on sucrose-containing medium (Figure 1A). TAP-Irc20 was strongly overexpressed relative to endogenous Irc20, producing a protein that migrates at the expected size of ∼200 kDa in SDS-polyacrylamide gels.

To further determine whether *IRC20* might have a role in transcription, we investigated whether Irc20 overexpression also exhibits an Spt- phenotype (Suppressor of Ty insertions). Spt- mutations alter transcription from the *lys2–128δ* and *his4–912δ* reporters that contain transposon insertions in each of their promoters [42]; the transposon insertions cause defective transcription of *LYS2* and *HIS4* respectively, causing Lys- and His- phenotypes, and *spt-* mutations restore correct transcription at *lys2–128δ* and *his4–912δ*, resulting in Lys+ and His+ growth. Expression of *IRC20* from its endogenous promoter on a 2 μ plasmid or from an integrated *GAL1* promoter was insufficient for activating either reporter. Expression of *IRC20* from the *GAL1* promoter on a 2 μ plasmid, however, was able to activate *lys2–128δ* (Figure 1B), but not *his4–912δ* (data not shown). Because overexpression sometimes can mimic a loss-of-function phenotype [43], we tested whether deletion of *IRC20* causes these transcription-related phenotypes, but *irc20Δ* strains were both Bur+ and Spt+ (data not shown). Elevated expression of Irc20 therefore is capable of producing more than one transcription-associated phenotype, presumably mediated by a gain of function.

To detect the expression level of Irc20 in the absence of the bulky TAP tag, a 6× hemagglutinin (HA) epitope tag was fused to the Irc20 C-terminus. Although full-length protein was detected, the 6xHA tag abolished the 2 μ *IRC20* Bur- phenotype (Figure 1C) indicating that this tagged fusion was non-functional. A 3xHA epitope tag was fused to the Irc20 N-terminus and full-length protein was again detected. In contrast to the C-terminal 6xHA-tagged fusion, the 2 μ N-terminal 3xHA-*IRC20* fusion was still Bur- (Figure 1C). These results indicated that the C-terminal 6xHA tag, but not the N-terminal 3xHA tag or N-terminal TAP tag, interfered with Irc20 function. N-terminal 3xHA-tagged or TAP-tagged Irc20 was used for all of the subsequent experiments in this report.

To further understand the mechanism responsible for the *IRC20* overexpression phenotype, we tested whether its two predicted protein domains were required. A conserved Snf2-family ATPase domain Walker B motif, which is a feature of many chromatin remodeling proteins [38], is located between amino acids 530 and 535. The Walker B motif contains adjacent conserved aspartic acid and glutamic acid residues that are required both for function *in vivo* [44] and for ATP-hydrolysis *in vitro* [45] in other Snf2-family ATPases. A *suc2Δuas(−1900/−390)* strain transformed with the 2 μ *irc20*-*D534A, E535A* ATPase mutant was incapable of growing on sucrose medium, indicating that the ability to hydrolyze ATP was necessary for the 2 μ *IRC20* Bur- phenotype (Figure 1D). Additionally, a C3HC4 RING finger domain, which is a defining feature of a subset of E3 ubiquitin ligases, is located between amino acids 1239 and 1277 of Irc20. Previous studies of other C3HC4 E3s demonstrated that mutation of the first of seven conserved cysteine residues that define this domain affect function *in vivo* and E3 ligase activity *in vitro* [45]. Overexpression of an *irc20-C1239A* RING mutant was incapable of producing the Bur- overexpression phenotype (Figure 1D). The lack of function of the ATPase and RING mutants was not due to trivial expression defects, as both mutants were expressed at equivalent levels to wild-type Irc20 (Figure 1D). Thus, *IRC20* required both its ATPase and RING finger activities to produce the high-copy Bur- phenotype, further suggesting that the high-copy phenotype is due to a gain of function, not via a dominant negative mechanism.

### *IRC20* has ubiquitin E3 activity

**Figure 2:**
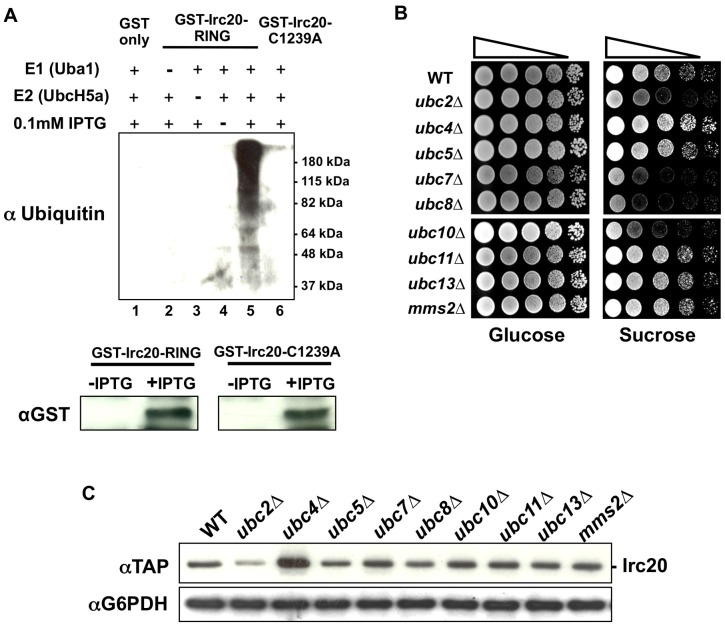
*IRC20* encodes an E3. A) Irc20 *in vitro* ubiquitylation assay. Reactions contained purified Uba1 (E1), UbcH5a (E2), ubiquitin, and bacterial lysates expressing either GST-Irc20 RING (pAR6), GST-Irc20 RING-C1239A (pAR21), or GST only (pGEX-KG). Reactions were incubated at 50°C for 90 minutes, subject to electrophoresis on SDS-PAGE, processed for western blot analysis, and probed with an anti-Ub antibody. 10 μg of total bacterial lysates from uninduced (“-IPTG”) and induced (“+IPTG”) cultures were analyzed by western blot to examine GST-Irc20 RING and GST-Irc20 RING-C1239A protein levels. B) E2 requirement for the *IRC20* overexpression Bur- phenotype. A 2 μ *TAP-IRC20* plasmid (pAR41) was transformed into a panel of nine non-essential ubiquitin E2 deletion strains and a spot test was performed on YPsucrose to assay the Bur- phenotype. C) Protein lysates were prepared from the E2 deletion strains in panel B transformed with pAR41 (2 μ *TAP-IRC20*) and Irc20 protein levels were examined by western blot analysis. G6PDH serves as a loading control.

Because the Irc20 RING domain was required for the Bur- overexpression phenotype, we next investigated whether *IRC20* possesses E3 activity *in vitro*. Attempts to purify soluble GST-Irc20 RING fusion protein (amino acids 1118 to 1556) from bacteria and from baculovirus-infected insect cells were unsuccessful, so crude bacterial lysates expressing GST fusions to wild-type Irc20 RING or Irc20-C1239A RING were assayed for E3 activity. In the presence of an E1 (yeast Uba1), an E2 (human UbcH5a), ubiquitin, ATP, and the extract expressing GST-Irc20 RING, a high molecular weight smear representing poly-ubiquitin chains of varying length was detected (Figure 2A lane 5). These ubiquitin chains were not observed in the absence of the E1 (lane 2) or E2 (lane 3), or when uninduced bacterial lysates (lane 4) or lysates expressing unfused GST (lane 1) were added to the reaction. Furthermore, E3 activity was not detected in the lysate expressing the Irc20-C1239A RING domain mutant that abolished the *IRC20* overexpression Bur- phenotype (lane 6). The lack of E3 activity for the RING mutant is not due to trivial expression issue, as Western blotting revealed equivalent expression to the wild-type protein (Figure 1A). These results indicated that the Irc20 RING domain possesses intrinsic E3 activity and suggested that Irc20 functions *in vivo* as an E3.

RING domain E3s recruit their substrates to an E2 to mediate the transfer of activated ubiquitin onto the substrate. Accordingly, the E2-E3 interaction is required for E3-mediated ubiquitylation. We reasoned that if Irc20 functions as an E3 *in vivo*, then deletion of genes encoding E2s that interact with Irc20 would block ubiquitylation of Irc20 substrates and suppress the 2 μ *IRC20* Bur- phenotype. 2 μ *IRC20* plasmids were transformed into a panel of the nine viable yeast ubiquitin E2 deletion strains and tested for whether any E2 deletions suppress the 2 μ *IRC20* Bur- phenotype (Figure 2B). Deletion of *UBC2, UBC7, UBC8*, and *UBC10* each suppressed the Bur- phenotype of 2 μ *IRC20*. Irc20 was not overexpressed well in the *ubc2Δ* strain, suggesting that suppression by *ubc2Δ* might simply be due to reducing Irc20 expression. In contrast, Irc20 protein levels in *ubc7Δ, ubc8Δ*, and *ubc10Δ* strains were similar to levels in the wild-type strain, ruling out diminished Irc20 levels as a reason for suppression (Figure 2C) and suggesting that these E2s directly or indirectly cooperate with Irc20 in the ubiquitylation pathway. Deletion of *UBC7*, *UBC8*, or *UBC10* did not reverse the Bur- phenotype of multiple genomic *bur* mutations, suggesting that these effects are specific for suppressing 2 μ *IRC20*. Taken together, these data support the idea that Irc20 acts in the ubiquitin pathway as an E3 and that this activity is important for its function.

### Identification of Irc20 physical interactions: Cdc48

**Figure 3:**
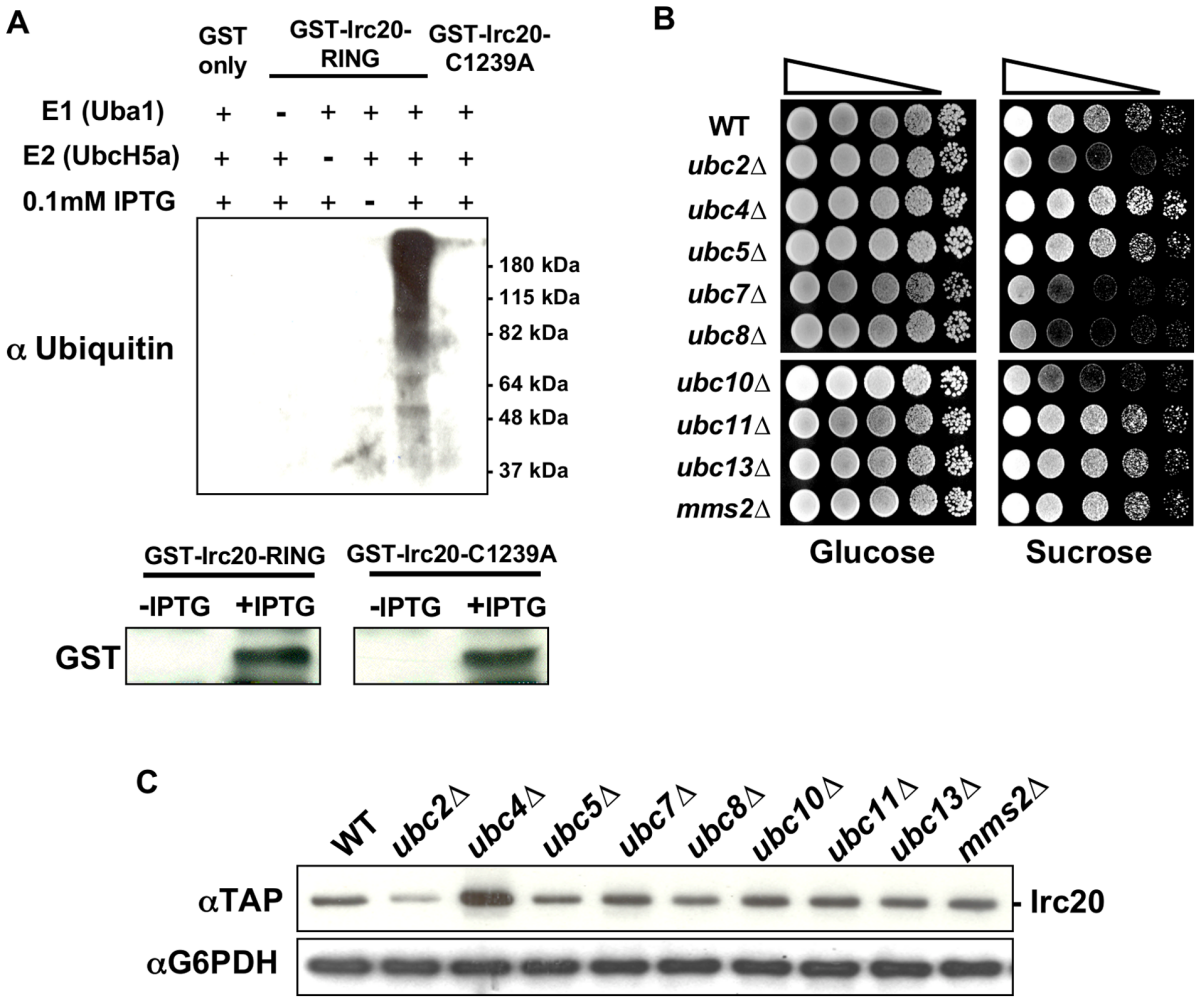
Irc20 physically interacts with Cdc48. A) Irc20 physical interactors from mass spectrometry. All proteins shown here were detected at levels more than 5-fold higher in the HA-Irc20 (pAR11) immunoprecipitations than in the untagged control (pGM7) immunoprecipitations (p≤0.055). B) Co-immunoprecipitation of Cdc48–3xMyc by HA-Irc20 and HA-Irc20 ATPase and RING mutants. Strains expressing either HA-Irc20 (pAR11) or its ATPase (pAR30) and RING- (pAR14) derivatives from 2 μ plasmids were harvested, extracts prepared, and immunoprecipitated with anti-HA agarose beads. Western blots of the eluted proteins were probed with either anti-HA (top panels) or anti-Myc (bottom panels) antibodies. WCE  =  crude whole cell extracts.

Deletion of *IRC20* does not cause any readily detectable plate phenotype in our strains, making it difficult to probe its function using traditional loss-of-function or suppressor genetics. In an effort to understand its function or regulation, we utilized two co-purification and mass spectrometry approaches to identify proteins that physically interact with Irc20. In the first approach functional N-terminal 3xHA-Irc20 (Figure 1C) was immunoprecipitated, non-specific proteins were washed away, and the eluted proteins processed for tandem mass spectrometry. A ratio of total spectral counts was obtained for each protein detected in co-immunoprecipitations (co-IPs) from three 3xHA-Irc20 replicates compared to the total spectral counts detected for each protein in untagged control co-IPs. Thirty-six proteins that had spectral counts enriched by at least 5-fold in the HA-tagged co-IPs compared to the untagged control (p≤0.055) were considered candidates for further experiments (Figure 3A and Table S1).

Preliminary tests to detect association of tagged versions of these candidate Irc20-interacting proteins revealed reproducible co-IP of Irc20 with Cdc48–3xMyc. Cdc48 is a conserved, essential AAA-ATPase that was enriched approximately 6-fold higher in the HA-tagged co-IPs compared to the untagged co-IPs. It is believed to function primarily as a segregase that extracts ubiquitylated proteins from protein complexes and membranes [46], thereby targeting ubiquitylated proteins for proteasome-mediated degradation [11]. Notably, Cdc48 associates with multiple E3s [10], [20] and has been implicated in regulating transcription factors, including the processing of Spt23 and mediating the UV-induced degradation of the largest subunit of RNA Pol II [16], [26]. To test the reproducibility of the mass spec results, Cdc48 was tagged at its C-terminus with 3xMyc. Cdc48–3xMyc restored viability of *cdc48Δ* strains, grew well, and was Bur+, indicating that it is functional, and the Cdc48–3xMyc protein co-immunoprecipitated equally well with wild-type Irc20 and with the Irc20-C1239A RING domain and Irc20-D534A, E535A ATPase domain mutants (Figure 3B). These results confirm that Cdc48 and Irc20 physically interact with each other and suggested that these two proteins might function together or that one protein might regulate the other.

### Identifying the region of Irc20 that is sufficient for binding Cdc48

**Figure 4:**
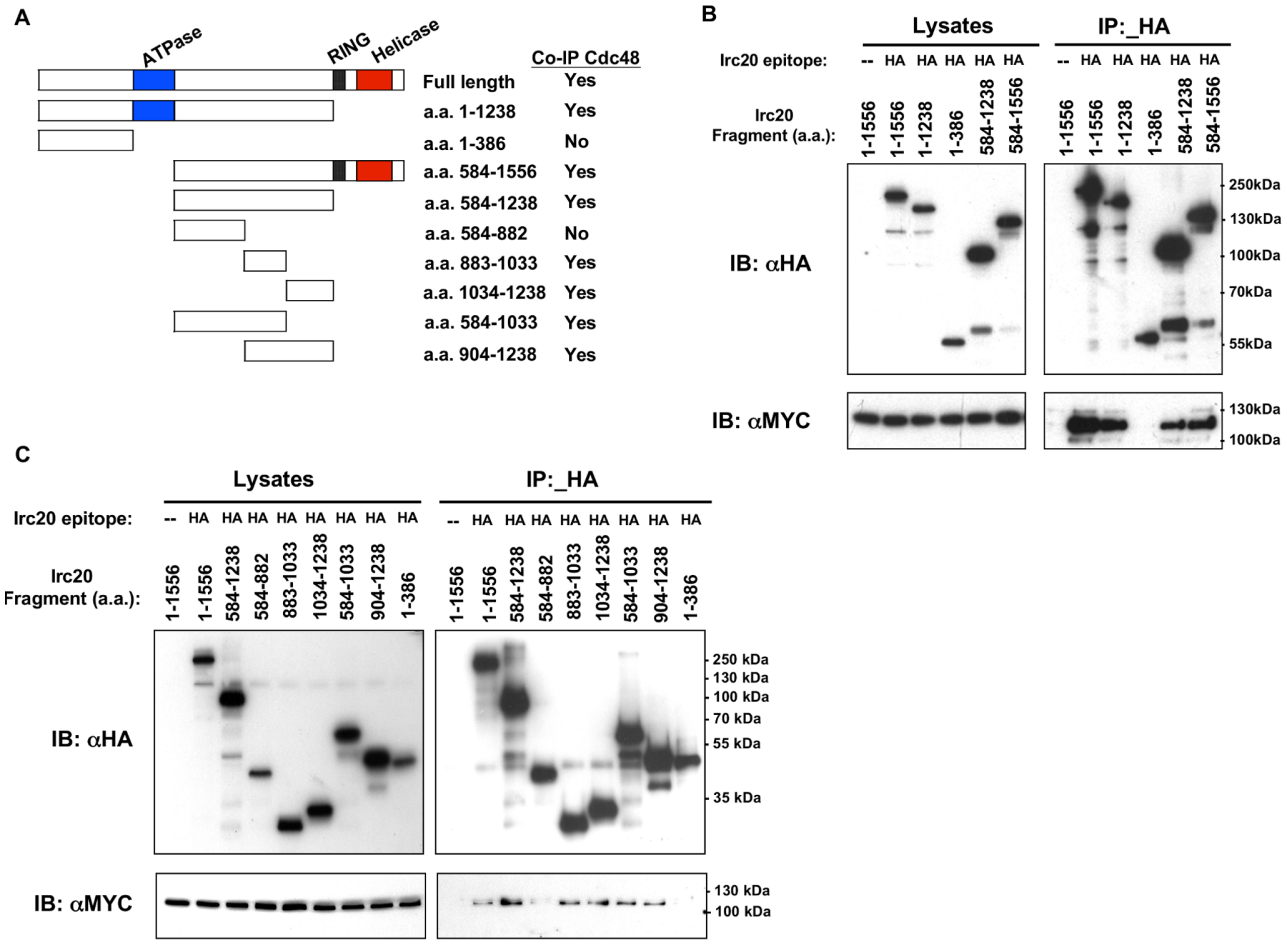
Identifying the Cdc48-binding domain of Irc20. A) Diagram of Irc20 deletion derivatives and a summary of their ability to co-IP Cdc48. B) & C) Co-immunoprecipitation analysis. The indicated Irc20 derivatives were expressed from a 2 μ plasmid in the presence of Cdc48–3xMyc, extracts were prepared, proteins immunoprecipitated with anti-HA beads, and western blotted with anti-HA antibody to detect Irc20, or with anti-Myc antibody to detect Cdc48. The binding interface of Irc20 that physically interacts with Cdc48 lies between amino acids 883 and 1238.

The Irc20 protein sequence does not contain any of the known Cdc48-interacting motifs that have been identified in other Cdc48-interacting proteins [19]. To define the region of Irc20 that was needed to co-IP with Cdc48, a series of 3xHA-tagged deletion derivatives that spanned the entire length of Irc20 was created (Figure 4A). From the first round of deletions designed to broadly localize the interacting domain, an N-terminal fragment of Irc20 (amino acids 1–386) was unable to co-IP Cdc48, while the derivatives that were able to co-IP Cdc48 contained amino acids 584–1238 (Figure 4B), pointing towards the importance of an internal Irc20 domain. Indeed, expression of Irc20 584–1238 was sufficient to co-IP Cdc48 (Figure 4B). To define the Cdc48-binding region further, smaller N-terminal HA-tagged deletion derivatives of the Irc20 584–1238 domain were created (Figure 4A) and tested for their ability to co-IP Cdc48 (Figure 4C). The Irc20 region spanning amino acids 584–882 did not co-IP Cdc48, but surprisingly, two adjacent non-overlapping regions (a.a. 883–1033 and a.a. 1034–1238) each were capable of co-immunoprecipitating Cdc48 (Figure 4C), suggesting that Cdc48 contacts Irc20 within both of these fragments. We conclude that the region of Irc20 between amino acids 883 and 1238 is required and sufficient for binding Cdc48 and that Cdc48 is recruited either directly or indirectly by different regions within this domain.

### Characterization of Irc20 mutants defective for binding Cdc48

**Figure 5:**
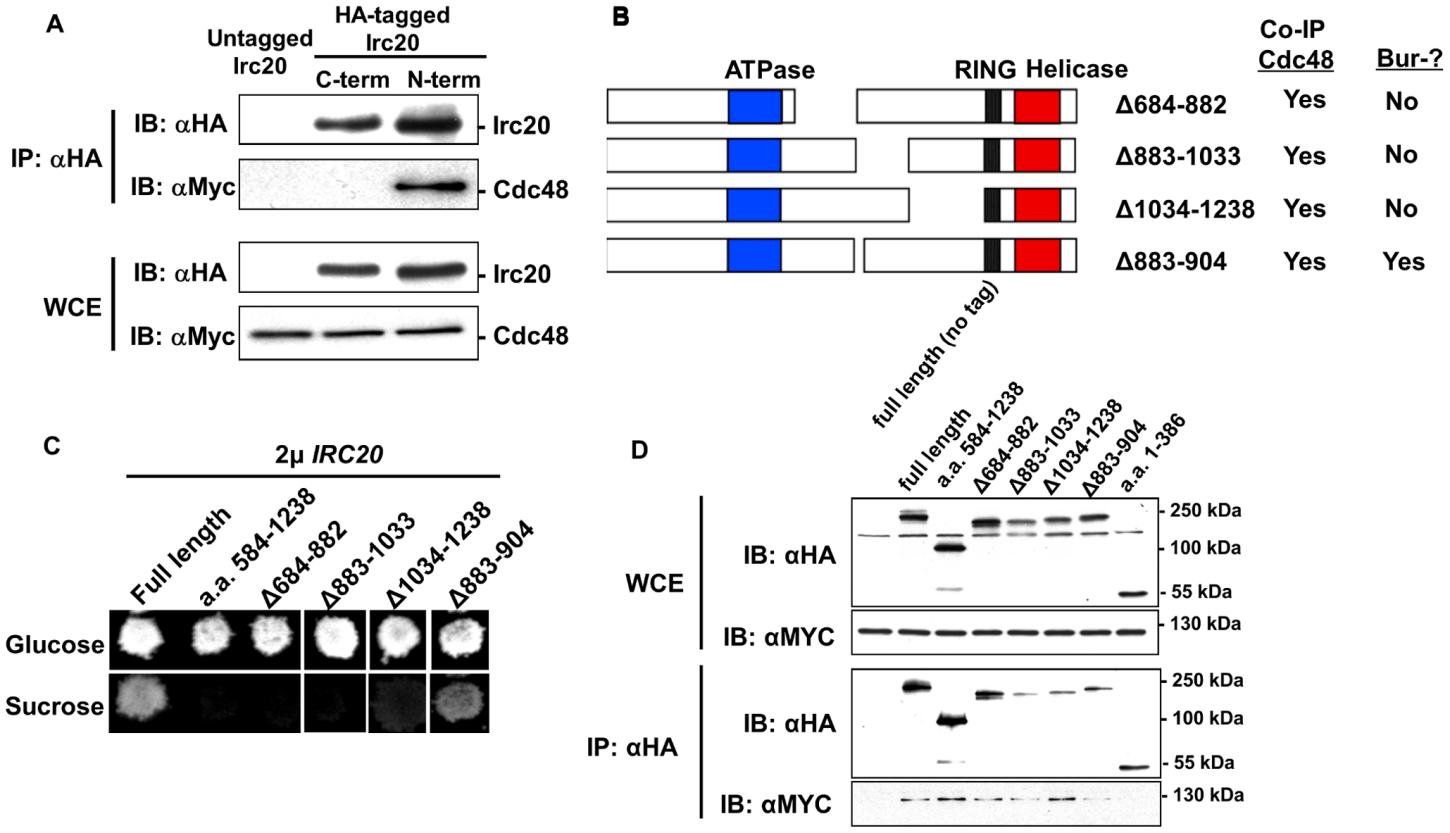
Characterization of *irc20* mutants defective for binding Cdc48. A) N-terminal (pAR11) and C-terminal (pAR9) 3xHA-Irc20 were expressed from a 2 μ plasmid in the presence of Cdc48–3xMyc, extracts were prepared, proteins immunoprecipitated with anti-HA beads, and western blotted with anti-HA antibody to detect Irc20, or with anti-Myc antibody to detect Cdc48. B) Diagram of deletions created with the context of full-length 3xHA-Irc20. A summary of their results in the functional assays and co-IPs presented in panels C and D is shown to the right. C) The *3xHA-irc20* deletion derivatives were expressed from a 2 μ plasmid and tested for their Bur- phenotype and D) for their ability to co-precipitate Cdc48–3xMyc. WCE  =  whole cell extract

Having identified a physical interaction between Irc20 and Cdc48 and defined a region necessary for this interaction, our next goal was to determine if this interaction was relevant for *IRC20* or *CDC48* functions. Using the C-terminal and N-terminal HA-tagged Irc20 derivatives that were described in Figure 1C in co-IPs, we found that functional N-terminal 3xHA-Irc20 co-immunoprecipitated Cdc48, but the non-functional C-terminal Irc20–6xHA did not (Figure 5A), suggesting that the Irc20-Cdc48 interaction might be required for the *IRC20* Bur- overexpression phenotype and for *IRC20* function.

Having obtained evidence from the C-terminal Irc20–6xHA tag experiment that the Irc20-Cdc48 interaction might be important, we next tested whether the Cdc48-interacting region of Irc20 was important for its overexpression phenotype. The regions of *IRC20* that were sufficient for Cdc48 interaction were deleted in the context of full-length *IRC20* (Figure 5B) and tested for their ability to cause the high copy Bur- phenotype and for the ability to co-IP with Cdc48. Unlike the Irc20 fragments used in Figure 4, these deletion derivatives carry both the RING finger and ATPase domains that are required for the high copy Bur- phenotype, allowing assessment of the importance of the Cdc48-interacting region. Deletion of the interacting fragments caused loss of the high copy Bur- phenotype (Figure 5C), but none affected the ability of Irc20 to co-IP with Cdc48 (Figure 5D), likely due to the redundancy of the interacting surfaces. We conclude that either the entire Cdc48-interacting region is required for function, or the deletions indirectly affect other functions of Irc20, such as its ATPase or E3 activity.

### *cdc48* mutations can cause a Bur- phenotype

**Figure 6:**
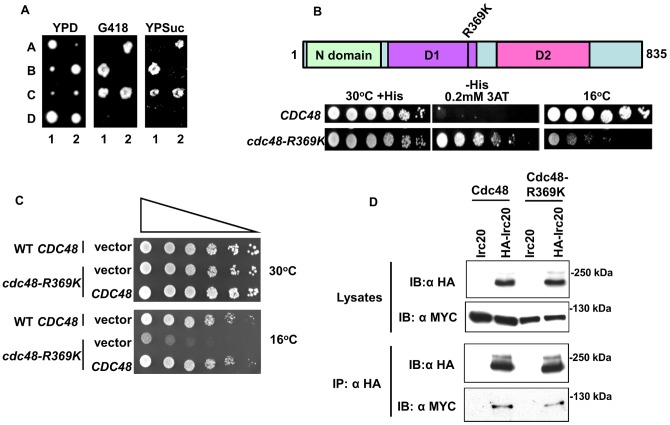
*cdc48* mutations can cause a Bur- phenotype. A) A heterozygous *CDC48–12xMYC::KAN* integrant diploid (GY2476) was induced to undergo meiosis and tetrads dissected. Two representative tetrads are shown, with spores labeled A-D. Perfect linkage was observed between slow growth, G418-resistance, and the Bur- phenotype in 30 four-spored tetrads. B) A plasmid shuffle screen for *cdc48* Bur- mutants uncovered an arginine-to-lysine change at amino acid 369, located in the ATPase D1 domain. This mutation confers both Bur- and cold-sensitive phenotypes (bottom panel). The strain being tested here contains the *suc2Δuas-HIS3* reporter, with growth on SC-His plate being indicative of the Bur- phenotype. C) The cold sensitivity of *cdc48-R369K* is complemented by *CDC48* on a CEN plasmid. Strains GY2387 (*cdc48Δ::TRP1* <pAR54  =  CEN *URA3 CDC48*> and GY2406 (*cdc48Δ::TRP1* <pAR64  =  CEN *URA3 cdc48-R369K*>) were transformed with empty vector or wild-type *CDC48* on a *LEU2*-marked CEN plasmid (pAR56). Cold sensitivity was assayed at 16°C. D) The *cdc48-R369K* mutation does not affect the ability of Cdc48 to co-immunoprecipitate with Irc20.

During the process of creating tagged Cdc48 strains, an attempt was made to integrate a 9xMYC epitope tag at the C-terminus of genomic *CDC48*. Tetrad dissection from one of the heterozygous *CDC48–9xMYC::KANMX4* diploid integrants unexpectedly displayed 2∶2 segregation of a Bur- phenotype that was very tightly linked to G418 resistance, with no recombinants in 30 tetrads (Figure 6A). Thus, the C-terminal tagging of Cdc48 resulted in a Bur- phenotype, similar to overexpression of its binding partner *IRC20*. Sequence analysis of the *CDC48* locus from the Bur- integrant revealed that the epitope tag elongated to 12xMYC, presumably from a recombination event mediated by the repetitive MYC sequence. The Bur- phenotype was unique to the 12xMyc tag, as strains expressing either a C-terminal Cdc48–3xMYC fusion or a bulkier C-terminal Cdc48-TAP tag fusion as the only source of Cdc48 were both Bur+. Unfortunately, the 12xMYC tag was highly unstable, as single colonies isolated from the original integrant rapidly yielded a mixed population of Bur- and Bur+ integrants carrying MYC epitopes of reduced and varying lengths, making it impossible to perform interpretable experiments with the 12xMYC strain. These results, however, were encouraging as they indicated that it might be possible to isolate Bur- *cdc48* mutants. Using a standard plasmid shuffle strategy [47], a library of randomly mutagenized *cdc48* mutants was created on a CEN plasmid and screened in a strain carrying a modified *suc2Δuas(−1900/−390)* reporter in which the *SUC2* open reading frame was replaced by *HIS3*. Increased transcription from the resulting *suc2Δuas(−1900/−390)-HIS3* reporter allowed us to assay the Bur- phenotype by selecting for growth on glucose-containing media lacking histidine. Two Bur- *cdc48* mutants were isolated, each of which carried the same arginine-to-lysine substitution at position 369 (Figure 6B). Arginine is highly conserved at this position within the second region of homology (SRH) in the D1 ATPase domain of AAA-ATPase family members [48], [49], with the orthologous residue in mammalian p97 being required for maintaining its hexameric structure and for the ability to bind ubiquitin chains [49]. Other *cdc48* mutant alleles (*cdc48–2, cdc48–3*, and *cdc48–10*) were crossed into the *suc2Δuas(−1900/−390)-HIS3* reporter background, but none of them were capable of growing on media lacking histidine, indicating that the Bur- phenotype is not the result of a general *CDC48* defect. The *cdc48-R369K* mutation confers a strong cold-sensitive phenotype that was complemented by a *CDC48* CEN plasmid, indicating that it is recessive and therefore likely due to a reduction of *CDC48* function (Figure 6C). Co-IP experiments (Figure 6D) demonstrated that the physical interaction between Irc20 and Cdc48 was unaffected by this mutation. Taken together with the results described above, either overexpression of *IRC20* or a loss-of-function mutation in its binding partner Cdc48 caused a Bur- phenotype.

### Identification of Irc20 physical interactions: SUMO

**Figure 7:**
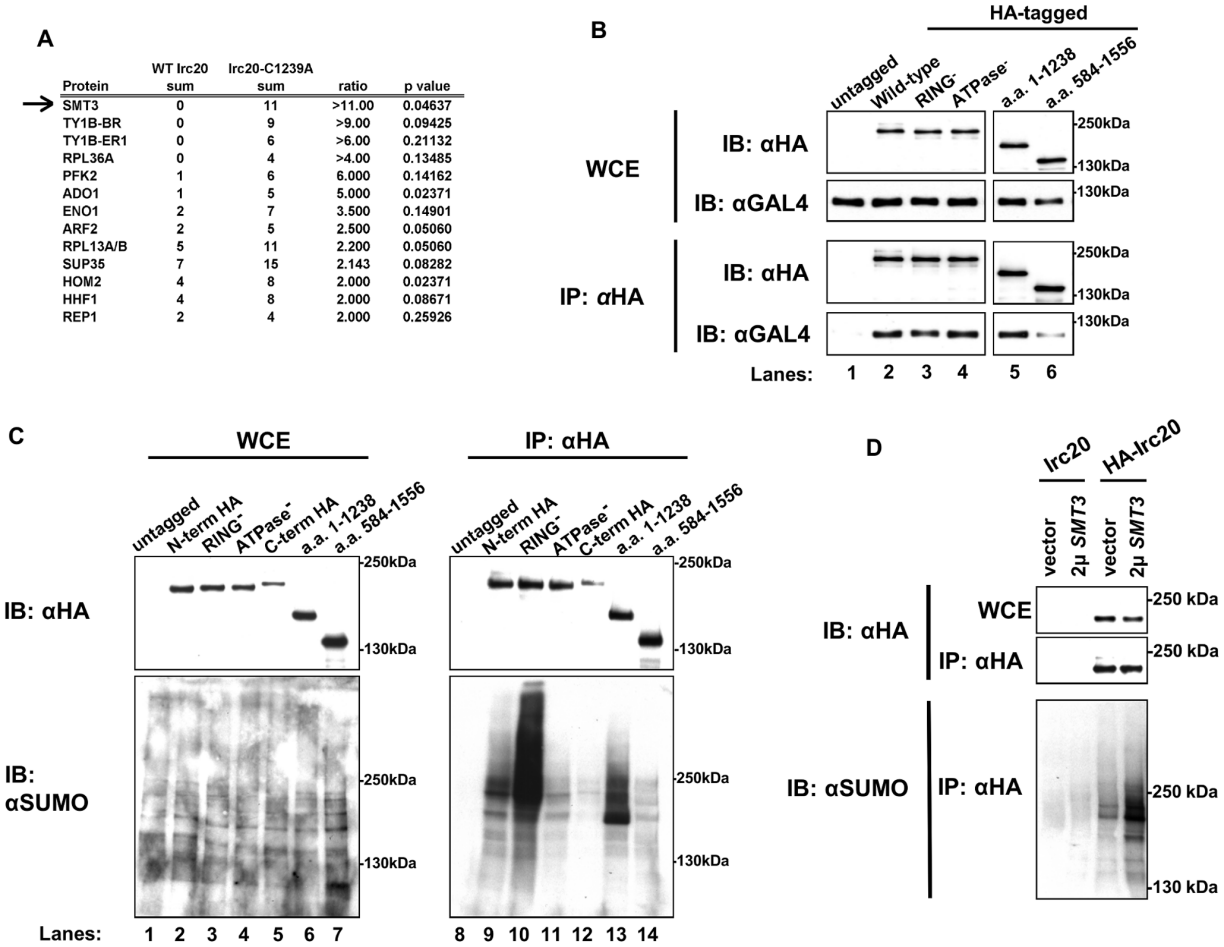
Irc20 is SUMOylated and binds SUMO. A) Proteins that preferentially associate with the irc20-C1239A RING- mutant. GY460 (*his4–912δ lys2–128δ suc2Δuas(−1900/−390) ura3–52 leu2Δ1*) expressing either pAR11 (*3xHA-IRC20*) or pAR14 (*3xHA-irc20-C1239A*) was cross-linked *in vivo* with formaldehyde, pelleted, lysed, immunoprecipitated, and the resulting material analyzed by mass spectrometry. All proteins that were detected at least 2-fold greater in the Irc20 RING- mutant co-IPs compared to the WT Irc20 co-IPs are presented. Smt3 ( = SUMO) was detected in the Irc20 RING- mutant at 11-fold greater level than in the wild-type 3xHA-Irc20 control. B) Irc20 binds SUMO. To address whether Irc20 is binding to SUMO, co-IPs under native conditions were performed with WT HA-Irc20 and RING- mutant HA-Irc20 to test for the ability of each to bind a non-conjugatable GAL4(DBD)-6xSUMO chain fusion protein. Both WT and RING- mutant Irc20 pull down the 6xSUMO in equal amounts, ruling out the possibility that RING- mutant Irc20 is simply binding to more SUMO than WT Irc20 in the previous experiment. C) Irc20 is SUMOylated. To test whether Irc20 is SUMOylated, the indicated proteins were immunoprecipitated and Western blots probed with anti-HA and anti-SUMO antibodies. D) To determine the level of SUMOylated Irc20 when SUMO is overexpressed, untagged Irc20 (pGM7) and 3xHA-Irc20 (pAR11) were co-IPd from lysates prepared from cell expressing only endogenous SUMO (“vector”) or overexpressing SUMO from pZW61 (2 μ *SMT3*). Western blots were probed with anti-HA and anti-SUMO antibodies.

Ultimately, to understand the roles of the Irc20 E3 *in vivo*, it is necessary to identify its substrates. Identifying substrates of E3s has proven to be particularly challenging, but one promising approach is to detect proteins that preferentially associate with a RING mutant version of the E3 [41]. The *irc20-C1239A* RING mutant was used in this approach with the hope that it would stabilize the ordinarily transient interaction between E3 and substrate. Mutation of one of the conserved zinc-coordinating cysteine residues is believed to collapse the globular structure of the RING finger domain in other E3s by rendering it incapable of binding its cognate E2 [50], [51]. Disrupting this interaction is predicted to inhibit ubiquitylation of the substrate and block its proteolysis, enabling it to be detected more readily by mass spectrometry. We therefore prepared samples from *3xHA-IRC20* and *3xHA-irc20-C1239A* RING mutant strains that had been cross-linked with formaldehyde to increase detection of associated proteins (Table S2). Only thirteen proteins associated at greater than 2-fold increased levels with the Irc20 RING mutant (Figure 7A), but unexpectedly the most highly enriched protein was Smt3 (SUMO), which was present at 11-fold higher levels. Notably, Cdc48 also was identified in this second round of mass spectrometry but was detected in equivalent levels in the wild-type and mutant HA-tagged co-IP's (Table S2). The identification of SUMO was intriguing in light of Irc20′s physical association with Cdc48 and because Cdc48 binds SUMO both through its own SUMO-interacting motif (SIM) and indirectly though Ufd1 [52]. Furthermore, Cdc48 preferentially binds SUMOylated Rad52 and appears to limit unnecessary recombination events by interrupting the Rad51-Rad52 interaction that is involved in the formation of Rad51 DNA repair foci [53].

We considered two likely reasons why SUMO is detected at increased levels with mutant Irc20; either the RING mutant Irc20 binds to SUMOylated proteins, stabilizing the interaction due to catalytic inactivity as we predicted, or SUMO is preferentially conjugated to the Irc20-C1239A mutant. To address whether Irc20 binds to SUMO, *3xHA-IRC20* and the *3xHA-irc20-C1239A* RING mutant were expressed in the presence of a non-conjugatable Gal4DBD-6xSUMO fusion protein and tested for the ability to co-IP Gal4BD-6xSUMO. The C-terminal di-glycine motif of SUMO is required for its conjugation to substrates [54], and Hannich *et al*. [55] demonstrated that removal of the di-glycine motif ensures that two-hybrid interactions observed with Gal4BD-SUMOΔGG occurs through non-covalent binding. We constructed a Gal4BD-6xSUMO fusion that lacks any di-glycine motifs, ensuring that all physical interactions observed with this bait occur through non-covalent binding. Irc20 and Irc20-C1239A both co-immunoprecipitated Gal4BD-6xSUMO, but equally well (Figure 7B, lanes 2 & 3). Similar results were obtained using our 3xHA-Irc20 ATPase mutant, which co-immunoprecipitated Gal4BD-6xSUMO as well as both WT and RING mutant Irc20 (Figure 7B, lane 4). In an attempt to define the responsible domains, two Irc20 deletion derivatives were tested for their ability to co-immunoprecipitate 6xSUMO. Although both derivatives were able to co-immunoprecipitate 6xSUMO, the region between amino acids 1 to 1238 co-immunoprecipitated 6xSUMO at levels equivalent to wild-type Irc20, while the region between amino acids 584–1556 co-immunoprecipitated Gal4BD-6xSUMO less effectively than wild-type Irc20 (Figure 7B, compare lanes 5 & 6 with lane 2). Combined, these results indicate that Irc20 binds to SUMO, that the Irc20 RING mutant does not bind SUMO to a greater extent than wild-type Irc20, and that similar to other SUMO-binding proteins, Irc20 appears to contain more than one SUMO interacting motif, with a major determinant residing in the domain between amino acids 1–583 and at least one other localizing between amino acids 584–1238.

To determine whether Irc20 was SUMOylated, 3xHA-Irc20 and the 3xHA-Irc20-C1239A RING mutant were immunoprecipitated under native conditions, subject to electrophoresis in a denaturing SDS gel, and analyzed by western blotting with anti-HA and anti-SUMO antibodies. A strong anti-SUMO cross-reactive band was detected slightly higher than 180 kDa, above the position of unmodified Irc20 (Figure 7C, lane 9). To rule out the possibility that the cross-reactive band recognized by anti-SUMO antibody was a simply a SUMOylated protein that co-immunoprecipitated with Irc20, a deletion derivative of Irc20 (a,a, 1–1238) was expressed in place of full-length Irc20. When HA-Irc20–1–1238 was expressed, the SUMO cross-reactive band that migrates at the expected position for full-length Irc20 was no longer observed and a new SUMO cross-reactive band was instead observed at the position expected for the deletion derivative (Figure 7C, lane 13), suggesting that the bands recognized by anti-SUMO antibody are SUMOylated Irc20. Furthermore, the band increased in intensity when SUMO was overexpressed (Figure 7D). The intensity of the SUMOylated Irc20 signal increased in the *irc20-C1239A* mutant (Figure 7C, lane 10), explaining the increase of SUMO detected in the mass spectrometry analysis. Interestingly, the intensity increased in the Irc20 derivative that is deleted for the RING domain (a.a. 1–1238) (Figure 7C, lane 13) but did not increase in an *irc20* ATPase mutant (Figure 7C, lane 11), in the C-terminally 6xHA-tagged Irc20 fusion protein that is defective for the Bur- overexpression phenotype (Figure 7C, lane 12), or in an Irc20 deletion derivative that spans amino acids 584–1556 which carries a RING domain but not the ATPase domain (Figure 7C, lane 14). These combined results demonstrated that wild-type Irc20 and RING mutant Irc20 bound SUMO, both were SUMOylated *in vivo*, and that an increase in Irc20 SUMOylated relative to wild-type Irc20 was due to a defect in the RING domain.

## Discussion

Previous studies implicated *IRC20* as functioning in DNA repair and synthesis-dependent strand-annealing-mediated homologous recombination [35], [36], but its specific role in these processes remains largely unstudied. Here we demonstrate that Irc20 has E3 activity, present genetic results that implicate a role for *IRC20* in transcriptional regulation, and demonstrate physical interactions of Irc20 with Cdc48 and SUMO. Although the Bur- and Spt- phenotypes occur when *IRC20* is overexpressed, and not in an *irc20Δ* strain, we believe that they implicate a role for *IRC20* in transcription based on the successful history of the Bur and Spt selections and because both the Irc20 ATPase and RING domains are required for the high copy Bur- phenotype. The requirement for both of the identifiable domains indicates that the overexpression phenotype is not occurring by a dominant negative mechanism, and makes it likely that the overexpression phenotype reflects its normal physiological role. Irc20 undoubtedly functions as an E3 *in vivo* based on the presence of a consensus RING domain, detection of E3 activity *in vitro*, disruption of E3 activity and the 2 μ Bur- phenotype by RING substitution mutations, and suppression of the 2 μ *IRC20* Bur- phenotype by E2 deletions. Unlike most RING or U-box E3s, Irc20 also contains a Snf/Swi family ATPase domain, an arrangement shared with yeast Rad16, Rad5, and Uls1 and with human SHPRH, HLTF, and TTF2. Similar to Rad16, Rad5, and Uls1, ATPase activity is also needed for *IRC20* function [45], [56], [57], but it is not yet known for any of these proteins how these dual activities function together. For example, one might speculate that ATPase activity is required to present a substrate for the RING-bound E2, or that ubiquitin modification of a protein by the E3 results in a substrate that can be acted upon by the ATPase. Because other Snf/Swi family ATPases are involved in chromatin remodeling [38], it remains plausible that both the transcription and recombination phenotypes reported for *IRC20* are mediated via changes in chromatin structure.

As part of their involvement in the ubiquitin pathway, RING finger proteins associate with specific E2s to facilitate the transfer of ubiquitin onto a recruited substrate [5], [6]. Our results demonstrate that deletion of the E2s *UBC7, UBC8*, and *UBC10* each suppressed the 2 μ Bur- phenotype (Figure 2B), providing independent support that Irc20 is functioning in the ubiquitin pathway *in vivo*. It remains unclear how these E2 deletions suppress the Bur- phenotype, as current studies indicate that each E2 is involved in different cellular functions. We remain open to the idea that these E2s have roles in different, heretofore unknown processes, but cannot rule out the possibility that one or more of these E2 deletions are suppressing indirectly. Another possibility, however, is that Irc20 function is mediated through more than one E2, as previous studies with other E3s demonstrated that signaling can require the sequential involvement of multiple E2s. For example, the anaphase-promoting complex (APC) first requires Ubc4 to initiate the mono-ubiquitylation of target substrates and then Ubc1 to extend the pre-conjugated ubiquitin into K48-linked chains for mitotic APC functions [58]. Similarly, it is possible that each of the E2 deletions that suppress 2 μ *IRC20* are required for the phenotype by directing the ubiquitin chain assembly of a substrate at different stages of the process. This could be achieved by Irc20 directly interacting with all three E2s or by only interacting with one E2 while the other E2s are involved in further processing of the substrate.

In the process of characterizing its interactions with other proteins, we found that Irc20 physically interacts with the essential AAA-ATPase Cdc48 (Figure 3). Cdc48 associates with multiple co-factors, including the UBX domain family of proteins that recruit substrates and directly regulate Cdc48 [59], and with ubiquitin E3s or de-ubiquitylating enzymes that modulate ubiquitin chain length [10], [21]. Cdc48 has a well-studied role in protein quality control and it is not unreasonable to speculate that Irc20 is being subject to Cdc48-mediated quality control as an artifact of its overexpression. Indeed, included on the list of proteins identified by mass spectrometry are two other quality control proteins, Ydj1 and Hsc82 (Figure 3A). Our results, however, indicate that the physical interaction is not merely an artifact of a quality control system as C-terminally HA-tagged Irc20 is overexpressed equivalently to N-terminally tagged HA-Irc20, yet only the functional N-terminally HA-tagged Irc20 co-IPs Cdc48 (Figure 5A). This result eliminates the possibility that simple overexpression of Irc20 results in association with Cdc48 as only the functional version co-IPs Cdc48.

At least eight distinct Cdc48-binding motifs have been defined from these previously studied Cdc48 interactors [19]. We mapped the Irc20 domain between amino acids 883–1238 as sufficient for binding Cdc48 (Figure 4). This region does not share any similarities to other known Cdc48 interactors, raising the possibilities that Irc20 contains a novel Cdc48-binding motif or that the interaction with Cdc48 occurs through an intermediate. Furthermore, the Irc20-Cdc48 interaction appears to be functionally relevant because a C-terminal 6xHA tag that interferes with the Irc20 overexpression Bur- phenotype also disrupts its ability to co-immunoprecipitate Cdc48 (Figure 5A).

Given that the interaction between Irc20 and Cdc48 appears to be physiologically relevant, what is its relationship to the Bur phenotype? Genetic data from this study indicates that Cdc48 and Irc20 function in opposing manners, as an increase in Irc20 activity produces a Bur- phenotype while a recessive and presumably loss-of-function mutation in *CDC48* does the same (Figure 6). Three models can be envisioned to describe the relationship between Irc20 and Cdc48. The first model proposes that *IRC20* and *CDC48* function in separate pathways that independently influence *suc2Δuas(−1900/−390)* transcription. We consider this model unlikely based on the physical interaction detected between Irc20 and Cdc48, which implies a more direct functional relationship. A second model predicts that Cdc48 inhibits *suc2Δuas(−1900/−390)* transcription with Irc20 having an overall activating role by inhibiting Cdc48. This model is consistent with the antagonistic relationship between the two genes. An equally plausible model proposes that Irc20 functions as an activator of *suc2Δuas(−1900/−390)* transcription and that Cdc48 functions upstream as an Irc20 inhibitor. Under this model, Irc20 is normally kept inactive by Cdc48-mediated inhibition, but *IRC20* overexpression overwhelms Cdc48′s ability to inhibit its function. Likewise, the *cdc48-R369K* mutation might impair Cdc48′s ability to inhibit Irc20. In either case, increasing Irc20 activity by overexpression is sufficient for promoting transcription from the *suc2Δuas(−1900/−390)* reporter. Identifying the ubiquitylation substrate of Irc20 will undoubtedly shed significant light on the exact functional context within which it operates and reveal its relationship with Cdc48.

In the process of trying to identify the Irc20 ubiquitylation substrate by mass spectrometry, we found that Irc20 associates with SUMO and that it associates to a greater extent with an Irc20 RING mutant. Unexpectedly, Irc20 binds non-covalently to SUMO and is also covalently modified by SUMO. Analysis of Irc20 deletion derivatives suggested the presence of multiple SIMs in Irc20, with at least one located near the N-terminus of Irc20 between amino acids 1–583, and at least one other located between amino acids 584–1238. Indeed, amino acids 84–88 (VDIEI) of Irc20 conforms to the [ILV]-[DE]-[ILV]-[DE]-[ILV] SIM definition [60], and Irc20 contains a [VI]-[VI]-x-[VIL] class of SIM [61] between amino acids 657–660 (IIPL). Further experiments are required to determine if these regions of Irc20 are indeed necessary and sufficient for binding SUMO and whether the presence of multiple SIMs indicates a preference for binding SUMO chains as opposed individual SUMO monomers.

The increased SUMOylation of the Irc20 RING mutant might provide another clue to its function. Our demonstration that Irc20 associates with SUMO *in vivo* and has E3 activity *in vitro* raises two possibilities: either those activities are independent of each other, or they are functionally linked, with Irc20 serving as a SUMO-targeted ubiquitin ligase (STUbL). Because STUbLs typically target substrates for proteolytic degradation, their inactivation often leads to an increase in the levels of SUMO conjugates of their target substrates [62], [63]. Intriguingly, that is precisely what we observe for Irc20 itself: an Irc20 RING finger domain mutant results in an increase in SUMO-conjugated Irc20 (Figure 7D), consistent with Irc20 SUMO-dependent auto-ubiquitylation. Alternatively, the increased SUMOylation of the Irc20 RING mutant could be part of a quality control system. To convincingly distinguish between these models, substrates will need to be identified and *in vitro* assays required to test whether ubiquitylation by Irc20 occurs preferentially on SUMO-conjugated substrates.

## Supporting Information

Table S1 **Mass spectrometry to identify Irc20-associated proteins.** Three biological replicates of yeast strain GY460 expressing either untagged *IRC20* (pGM7) or *3xHA-IRC20* (pAR11) were subject to immunoprecipitation, and tandem mass spectrometry was performed on the eluted proteins. A ratio of total spectral counts was obtained for each protein detected in the three 3xHA-Irc20 replicates compared to the total spectral counts detected for each protein in untagged control immunoprecipitations, and t-tests were performed.
(XLS)

Table S2 **Mass spectrometry of cross-linked samples.** Three biological replicates of yeast strain GY460 expressing either untagged *IRC20* (pGM7), *3xHA-IRC20* (pAR11), or *3xHA-irc20-C1239A* (pAR14) were cultured, crosslinked with 1% formaldehyde, immunoprecipitated, and processed for mass spectrometry analysis as detailed in Materials and Methods. A ratio of total spectral counts was obtained for each protein detected in IPs from the three 3xHA-Irc20 replicates and the three 3xHA-Irc20-C1239A replicates compared to the total spectral counts detected for each protein in untagged control IPs. A ratio of total spectral counts was also obtained for each protein detected in IPs from the three 3xHA-Irc20-C1239A replicates compared to the total spectral counts detected for each protein in 3xHA-Irc20 IPs.
(XLS)
