# Correction: Physical and Genetic Associations of the Irc20 Ubiquitin Ligase with Cdc48 and SUMO

**DOI:** 10.1371/annotation/72c05c58-2c2b-4d3b-a9b4-20a46dba9c05

**Published:** 2013-11-14

**Authors:** Aaron Richardson, Richard G. Gardner, Gregory Prelich

The image currently appearing as Figure 3 is incorrect. Please view the correct Figure 3 here: 

**Figure:**
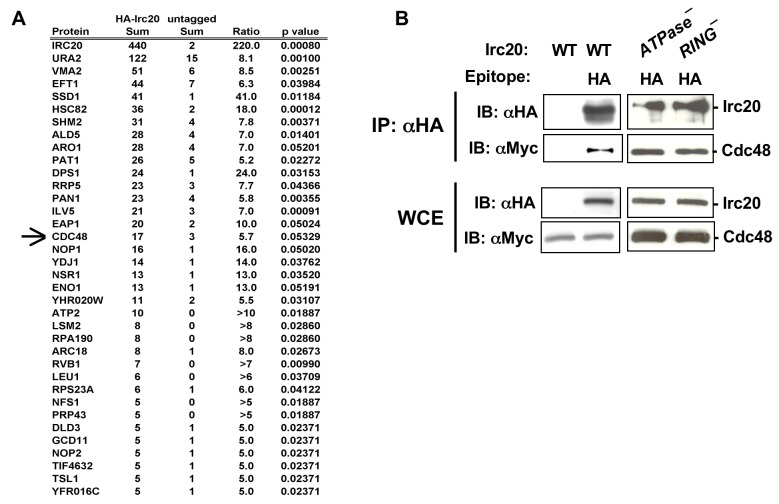


The title and legend for Figure 3 are correct as they are. 

